# Homogeneous Freezing of Water Using Microfluidics

**DOI:** 10.3390/mi12020223

**Published:** 2021-02-23

**Authors:** Mark D. Tarn, Sebastien N. F. Sikora, Grace C. E. Porter, Jung-uk Shim, Benjamin J. Murray

**Affiliations:** 1 School of Earth and Environment, University of Leeds, Leeds LS2 9JT, UK; sikora.scientific@gmail.com (S.N.F.S.); ed11gcep@leeds.ac.uk (G.C.E.P.); 2 School of Physics and Astronomy, University of Leeds, Leeds LS2 9JT, UK; J.Shim@leeds.ac.uk

**Keywords:** ice nucleation, homogeneous freezing, interfacial energy, water, droplet microfluidics

## Abstract

The homogeneous freezing of water is important in the formation of ice in clouds, but there remains a great deal of variability in the representation of the homogeneous freezing of water in the literature. The development of new instrumentation, such as droplet microfluidic platforms, may help to constrain our understanding of the kinetics of homogeneous freezing via the analysis of monodisperse, size-selected water droplets in temporally and spatially controlled environments. Here, we evaluate droplet freezing data obtained using the Lab-on-a-Chip Nucleation by Immersed Particle Instrument (LOC-NIPI), in which droplets are generated and frozen in continuous flow. This high-throughput method was used to analyse over 16,000 water droplets (86 μm diameter) across three experimental runs, generating data with high precision and reproducibility that has largely been unrepresented in the microfluidic literature. Using this data, a new LOC-NIPI parameterisation of the volume nucleation rate coefficient (*J*_V_(*T*)) was determined in the temperature region of −35.1 to −36.9 °C, covering a greater *J*_V_(*T*) compared to most other microfluidic techniques thanks to the number of droplets analysed. Comparison to recent theory suggests inconsistencies in the theoretical representation, further implying that microfluidics could be used to inform on changes to parameterisations. By applying classical nucleation theory (CNT) to our *J*_V_(*T*) data, we have gone a step further than other microfluidic homogeneous freezing examples by calculating the stacking-disordered ice–supercooled water interfacial energy, estimated to be 22.5 ± 0.7 mJ m^−2^, again finding inconsistencies when compared to theoretical predictions. Further, we briefly review and compile all available microfluidic homogeneous freezing data in the literature, finding that the LOC-NIPI and other microfluidically generated data compare well with commonly used non-microfluidic datasets, but have generally been obtained with greater ease and with higher numbers of monodisperse droplets.

## 1. Introduction

The freezing of water is of great importance in the glaciation of clouds, affecting their radiative properties and lifetime and in turn influencing the climate [1,2]. In the absence of nucleation sites, water is able to supercool to well below 0 °C before it freezes homogeneously, and clouds can be sensitive to homogeneous freezing at temperatures of −35 °C or even warmer [3,4]. A comprehensive understanding of the kinetics of the homogeneous freezing of water could enable more accurate representation of clouds and their glaciation in atmospheric models, but literature values and parameterisations of the volume nucleation rate coefficient, *J*_V_(*T*), of water, often based on the experimental freezing of purified water droplets [5,6], vary considerably [6,7]. In order to generate high-quality datasets to help constrain homogeneous freezing parameterisations, it is important to obtain freezing data using large numbers droplets with accurate measurements of their size and temperature. New instrumentation, such as that based on droplet microfluidic technology [8,9,10], could help to achieve this.

In the last decade or so, droplet microfluidic technology has been increasingly employed for ice nucleation analysis. Microfluidics enables the high-throughput generation and freezing of monodisperse micrometre-scale droplets in temporally and spatially controlled environments. Micrometre-scale droplets (pL volume range) avoid issues with impurities that typically hamper experiments with millimetre-scale droplets (e.g., μL volume) by triggering heterogeneous freezing at warmer temperatures [11,12,13], whilst also avoiding the high internal Laplace pressures associated with nanometre-scale droplets [14,15,16,17,18,19,20,21,22,23]. Further, microfluidic ice nucleation techniques employ droplets with diameters in the tens of micrometres, therefore being over the approximately 6 μm threshold above which nucleation is expected to occur in the volume of the droplet rather than at the surface [7,24,25].

Microfluidics can provide a simple and user-friendly route to performing monodisperse droplet freezing experiments, while more elaborate techniques can be taken advantage of to improve automation and throughput. As such, a number of microfluidic instruments have been applied to the freezing of droplets. These platforms can vary greatly in complexity, from the on-chip generation and off-chip freezing of water-in-oil emulsions [26,27,28], to the incorporation of on-chip droplet traps that allow the freezing of droplets in microarrays [29,30,31,32,33,34,35], and finally the generation and freezing of droplets in continuous flow [36,37,38,39]. However, only a handful of these have been applied to the study of homogeneous nucleation, while most have involved freezing pure water droplets as controls/backgrounds when performing measurements of ice-nucleating particles (INPs). Most instances of microfluidic water freezing have not demonstrated evidence of reproducibility, making the precision of the measurements unclear, whilst a number do not provide sufficient descriptions of their temperature calibration, which is highly important since temperature accuracy has been described as the most important uncertainty in *J*_V_(*T*) [26].

**Figure 1:**
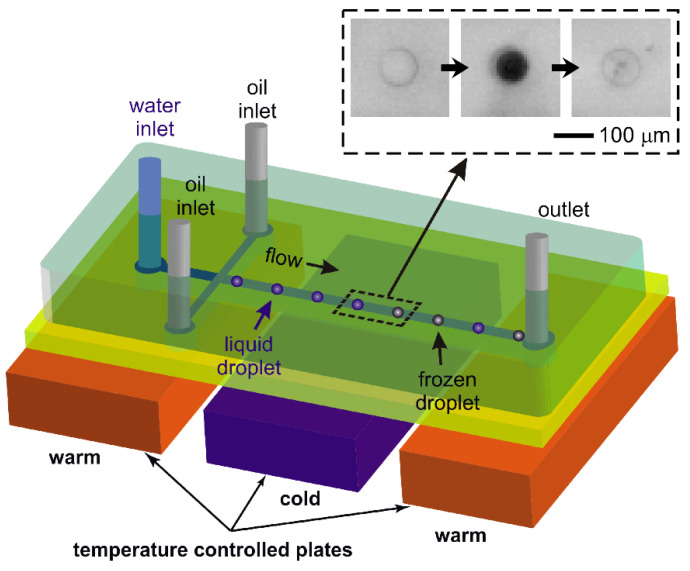
Schematic showing the core design and operation of the LOC-NIPI platform, in which water-in-oil droplets are generated and pass over a cold plate in continuous flow. A portion of the droplet population freezes as the droplets cross the cold plate, with the fraction of the droplets that freeze being dependent upon the temperature of the plate. The warm plates prevent freezing at the inlets and outlet and aid in reproducible droplet generation. The photographs inset show a single droplet freezing as it passes over the cold plate, changing from a colourless circle when liquid to a black circle once ice nucleation has initiated and the dendritic growth of ice has occurred, and finally to a near-colourless circle following full crystallisation to an ice crystal. The time between each photograph is 42 ms.

Recently, we demonstrated the development of the Lab-on-a-Chip Nucleation by Immersed Particle Instrument (LOC-NIPI) [39], a continuous flow platform in which droplets are generated in a heat transfer oil and flow over a cold plate at a series of subzero temperatures (Figure 1), whereupon the fraction of droplets that freeze at each temperature can be determined. The LOC-NIPI enables the measurement of hundreds or thousands of droplets per temperature set point in a short timeframe. Its design is also intended to allow the integration of other microfluidic components and techniques for downstream processing and analysis, as demonstrated by the recent incorporation of a feature for the sorting of water droplets and ice crystals in continuous flow [40].

Here, we take advantage of the high-throughput nature of the LOC-NIPI to analyse homogeneous freezing from over 16,000 water droplets (86 ± 8 μm diameter). The reproducibility and the precision of the technique were highlighted by collecting data from three experimental runs with slight variances in their operating parameters. We use this high-quality data to generate a new *J*_V_(*T*) parameterisation that we use to challenge a recent theoretical prediction [41] that is commonly used to represent *J*_V_(*T*) in the literature. Further, we briefly review and compile all of the available microfluidic *J*_V_(*T*) data available in the literature, comparing it with the LOC-NIPI and a selection of non-microfluidic datasets. Finally, we use classical nucleation theory (CNT) to estimate the interfacial energy of the stacking-disordered ice–supercooled water interface, *σ*_sd,l_, from the LOC-NIPI data, the first time this has been demonstrated in the microfluidics literature. We then apply this theory to the other available microfluidic *J*_V_(*T*) datasets, again comparing these and the LOC-NIPI data to more recent parameterisations and non-microfluidic datasets.

## 2. Materials and Methods

The LOC-NIPI water freezing data used in this manuscript were previously shown in Tarn et al. [39], which describes the microfluidic setup and the validation of the LOC-NIPI platform. Therefore, only a brief description of the LOC-NIPI and its operation is provided here, and the reader is directed to Tarn et al. [39] should they require further technical details. 

### 2.1. Chemicals

Purified water (18.2 MΩ cm at 25 °C, 0.22 μm filtered) was obtained from a Sartorius arium® pro water purification system. 3M™ Novec™ 7500 Engineered Fluid [42] is a fluorinated heat transfer oil that was used as the continuous phase for generating droplets, and was purchased from Fluorochem Ltd. (Hadfield, UK). Pico-Surf™ 1 (5% w/w in Novec™ 7500 oil) is a fluorinated surfactant [43,44] used for stabilising the droplets, which was purchased from Sphere Fluidics Ltd. (Cambridge, UK) and further diluted to 2% w/w in Novec™ 7500 oil for experiments. Silicone oil, used to form an interface between the microfluidic plate and the cold stage platform to improve heat transfer, was purchased from Sigma-Aldrich (Dorset, UK). Polydimethylsiloxane (PDMS, Dow Corning® Sylgard® 184 Kit) was purchased from Ellsworth Adhesives (East Kilbride, UK).

### 2.2. Microfluidic Chip Design and Fabrication

The design comprised two separate channel layouts: one for droplet generation and freezing, and one for providing an on-chip temperature measurement. The droplet generation and freezing layout incorporated a flow focussing nozzle (40 μm in width) for water-in-oil droplet generation that expanded into a main channel (300 μm in width, 31.5 mm in length) for freezing droplets as they flowed through the channel. This basic layout is shown schematically in Figure 1, while a detailed channel design layout can be found in Tarn et al. [39]. The temperature reference channel was located parallel to the droplet channel design, and featured an oil inlet and a long main channel with the same dimensions as in the droplet freezing layout. Into this channel would be inserted a thermocouple for measuring the on-chip temperature of the flowing oil, as a proxy for the temperature experienced by the droplets in the parallel droplet freezing channel.

The chip design was fabricated in PDMS using standard soft lithography techniques [45,46,47] to yield a channel height of 140 μm. The PDMS chips were bonded to glass microscope slides whose undersides were coated with a thin layer of chromium via metal evaporation, providing a mirrored surface that would enhance droplet visualisation during experiments. The PDMS and glass slides were bonded following treatment with oxygen plasma [46].

### 2.3. Experimental Setup

Polyethylene tubing (Smiths Medical, 0.38 mm i.d. (inner diameter) × 1.09 mm o.d. (outer diameter), Harvard Apparatus (Biochrom Ltd.), Cambridge, UK) was inserted into the oil and water inlet holes of the microfluidic device and attached at the other end to 1 mL syringes that were inserted into syringe pumps (PHD Ultra, Harvard Apparatus (Biochrom Ltd.), UK). The same type of tubing was inserted into the outlet holes of the chip and fed into a waste vial. A K-type thermocouple (80 μm diameter, ±2.2 °C, 5SRTC-TT-KI-40-1M series, Omega Engineering Ltd., Manchester, UK) was glued into a short section of polyethylene tubing, and was inserted into the temperature reference channel such that its tip was inside the channel itself, with the short piece of polymer tubing providing a seal with the PDMS chip. The thermocouple was connected to a data logger (TC-08, ±0.025 °C, Pico Technology, St. Neots, UK) for recording the on-chip temperature of the flowing oil with a 1 second time resolution. This measurement technique was carefully calibrated (see details in Tarn et al. [39]), yielding a measurement uncertainty of ±0.7 °C in the temperature range explored in this work (−35.0 to −36.9 °C).

The microfluidic chip was placed into the chamber of a custom-built cold stage, where it was situated over three temperature-controlled aluminium plates, with a thin layer of silicone oil added between the chip and the plates to aid heat transfer. Control of the plate temperatures was achieved using Peltier elements via a proportional–integral–derivative (PID) loop. The chip was situated such that the inlet channels and flow focussing nozzle were located over the first plate, a section of the main channel was located over the middle plate, and the outlet hole was situated over the third and final plate. The tip of the thermocouple in the temperature reference channel was located centrally over the middle plate, i.e., at the coldest region of the plate. The top of the cold stage chamber was covered with a sheet of Perspex to allow visualisation of the microfluidic chip. The chamber was also fitted with an inlet for dry air that allowed the chamber to be continuously purged of moisture and so avoid the formation of condensation, with the dry air exiting via outlet holes in the Perspex lid.

Visualisation of the droplets inside the microfluidic channel was performed using a Navitar Zoom 6000® lens system (Mengel Engineering, Denmark), with videos collected using a Phantom Miro Lab 120 high-speed camera (Vision Research Ltd., Bedford, UK). Videos were analysed using PCC 2.7 software (Vision Research Ltd.).

### 2.4. Experimental Procedure

With the chip placed over the three aluminium plates and the chamber closed using the Perspex lid, Novec™ 7500 oil (containing 2% w/w Pico-Surf™ 1) and purified water were pumped into the chip at flow rates of 22–24 μL min^−1^ and 0.02–0.05 μL min^−1^, respectively; the specific flow rates used for each experiment are provided in Table 1. Dry air was pumped into the chamber at 0.5 L min^−1^ in order to remove moisture and prevent the formation of condensation on the chip. The first and third temperature-controlled plates in the cold stage platform (i.e., the plates beneath the inlet channel structure and the outlet of the chip, respectively) were each set to +3 °C. The warm plate beneath the inlets ensured that the water would not freeze in the inlet channels or the nozzle, and that the droplet production would be reproducible since varying the nozzle temperature can change the droplet size and velocity [48]. The warm plate beneath the outlet ensured that any frozen droplets would be melted prior to exiting the chip, and would thus prevent any potential blockages from occurring due to a build up of ice crystals. The middle temperature-controlled plate was used as a cold plate in order to freeze droplets as they flowed through the main channel. This cold plate was set such that the temperature inside the chip was between −35–37 °C, as measured by the thermocouple inserted into the temperature reference channel. The setup of the chip and the three temperature-controlled plates is shown schematically in Figure 1, which also shows the generation and passing of droplets through the main channel, whereupon some of them freeze as they pass over the cold plate.

Once the main channel of the chip had reached the desired subzero temperature, videos of flowing droplets were collected for 100 to 200 s, which would later be used to determine the number of droplets that froze out of the droplet population as they moved through the channel. The inset photographs in Figure 1 show the freezing of a droplet as it passes over the cold plate, with the colourless liquid droplet becoming black after ice nucleation due to the dendritic growth of ice in the droplet, before fully crystallising to become a near-colourless ice crystal. The temperature was reduced incrementally by either 0.1 or 0.2 °C, with videos collected at each temperature interval, until reaching temperatures at which all of the droplets in the given timeframe were observed to freeze as they passed through the channel. The full experimental procedure was performed three times, and the experimental details for each run are provided in Table 1 in terms of the applied parameters (e.g., flow rates and temperature interval), the number of droplets analysed per temperature interval and in total, and the droplet sizes and volumes. Where relevant, the overall numbers for these different features are also provided in Table 1.

## 3. Results and Discussion

We first summarise the analysis of over 16,000 droplets during three experimental runs, before providing a new *J*_V_(*T*) parameterisation of the data that we compare to theoretical predictions. We further summarise *J*_V_(*T*) parameterisations obtained using other microfluidic platforms, alongside a selection of other available parameterisations and non-microfluidic datasets, to provide a comparison for our data and for microfluidic data as a whole. Finally, we estimate the interfacial energy of the ice cluster–supercooled water interface (*σ*_sd,l_) from our data and compare it to values we have calculated for other microfluidic publications and to a selection of other available literature data.

### 3.1. Homogeneous Freezing of Purified Water Using the LOC-NIPI

The results for each of the three experimental runs were obtained by counting the number of droplets that froze (*n*_ice_(*T*)) out of the total droplet population (*n*_tot_) at each temperature (*T*) interval. This allowed the fraction frozen (*f*_ice_(*T*)) to be calculated for each temperature interval using Equation (1) [11]:

(1) $$f_{\mathrm{ice}}\left(T\right)=\frac{n_{\mathrm{ice}}\left(T\right)}{n_{\mathrm{tot}}}$$

**Figure 2:**
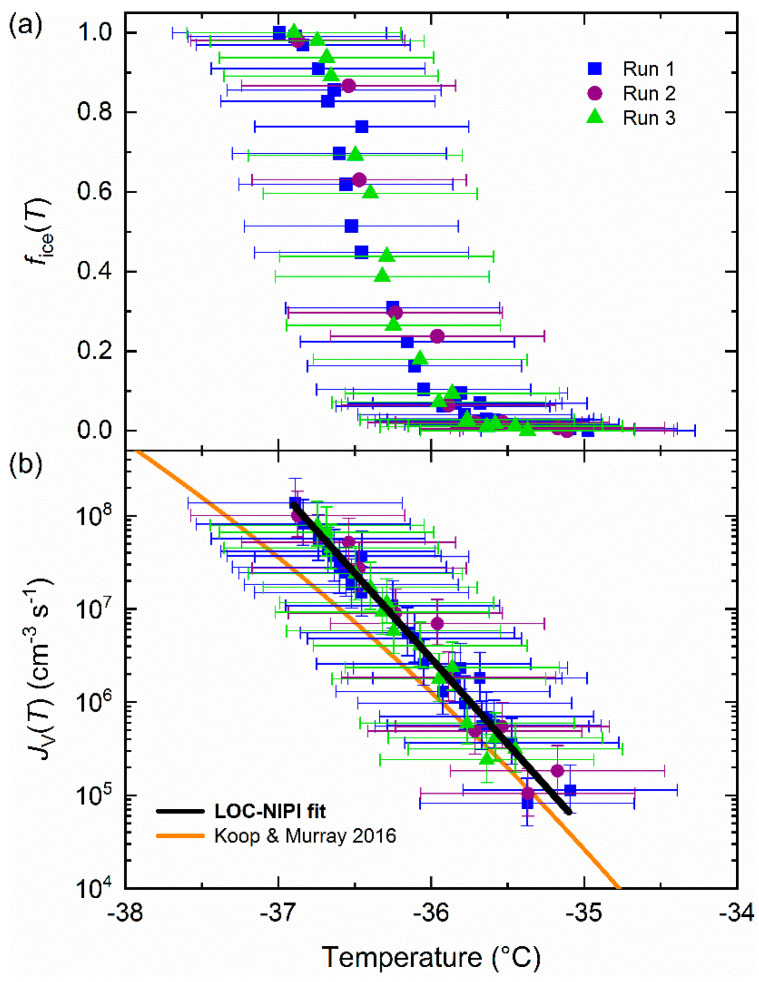
LOC-NIPI data for the homogeneous freezing of pure water droplets across three experimental runs: (**a**) The fraction frozen, *f*_ice_(*T*), of the droplet population; (**b**) the volume nucleation rate coefficient, *J*_V_(*T*), and overall fit of the data (see Table 2 for details of the applied fit), with the physically constrained CNT parameterisation of Koop and Murray [41] shown for comparison.

The *f*_ice_(*T*) values from each experimental run are shown in Figure 2a, and, importantly, demonstrate the reproducibility of the results even when using slightly different parameters (e.g., flow rates, temperature intervals, number of droplets analysed per temperature interval). Since each *f*_ice_(*T*) value is produced individually by the LOC-NIPI, *f*_ice_(*T*) can be related to the volume nucleation rate coefficient, *J*_V_(*T*) (cm^−3^ s^−1^), i.e., the number of ice nucleation events per unit volume per unit time, via Equation (2) [7,11,26]:

(2) $$J_{V}\left(T\right)=\frac{-\ln\;(1-f_{\mathrm{ice}}\left(T\right))}{V\;\Delta t}$$

where *V* is the droplet volume (cm^3^) and Δ*t* is the time interval (s) during which droplet freezing occurs. The values of *V* used here are provided in Table 1. The Δ*t* value was 0.13 ± 0.01 s, which was based on the amount of time that the droplets would spend over the coldest region of the cold plate (estimated to be a 1.5 ± 0.5 mm region at the centre of the cold plate), given their velocity. The *J*_V_(*T*) values from each experimental run are shown in Figure 2b, and the uncertainties in *J*_V_(*T*) were calculated from the combined uncertainties in the droplet volume and the time period. The uncertainty in droplet volume could be reduced in future experiments by using a greater magnification of the zoom microscope setup in order to provide higher resolution during the measurement of the droplet diameters. A conservative temperature uncertainty of ±0.7 °C was determined based on multiple calibration tests of the comparative temperatures between the droplet channel and the temperature reference channel, as detailed in Tarn et al. [39].

Experimental *J*_V_(*T*) values are often empirically approximated with the log-linear relationship shown in Equation (3) [7,25,26,49,50,51]:

(3) $$J_{V}\left(T\right)=e^{aT+b}$$

**Figure A1:**
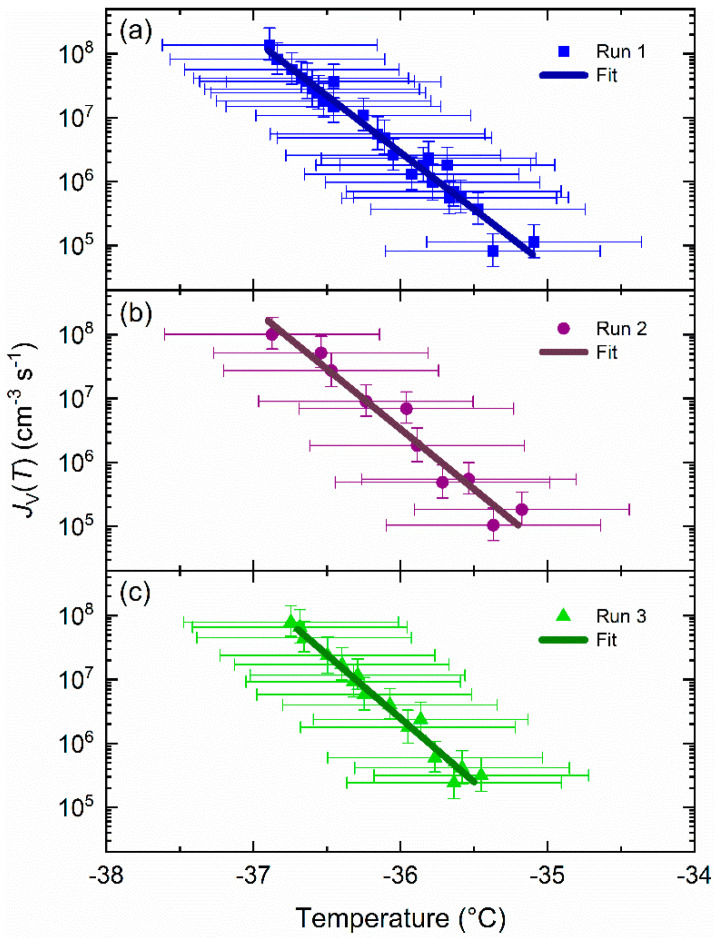
Plots showing the volume nucleation rate coefficients, *J*_V_(*T*), for the homogeneous freezing of purified water in each of the three microfluidic runs, together with their individual fits: (**a**) run 1; (**b**) run 2; (**c**) run 3. The experimental details of each run are provided in Table 1. The fit of *J*_V_(*T*) of each run, together with the R^2^ value of each fit, is provided in Table 2. The temperature uncertainty was estimated to be ±0.7 °C.

By plotting the data in terms of ln(*J*_V_(*T*)) vs. temperature *T* and applying a linear fit, the *a* and *b* coefficients are represented by the slope and intercept values of the fit, respectively. This method was applied to the *J*_V_(*T*) data shown in Figure 2b, both for the individual runs and for all of the data together, and the resultant fits and uncertainties are provided in Table 2. The *J*_V_(*T*) fits for each individual run are shown in Figure A1 of Appendix A, while the overall fit, determined by fitting to all of the data, is shown as a black line in Figure 2b. 

The overall fit for all of the data was ln *J*_V_(*T*) = −4.2171·*T* − 136.9602, in the temperature range of −35.1 to −36.9 °C. This is different to the equation originally reported for the LOC-NIPI in Tarn et al. [39], which was obtained by simply applying a linear fit to the *J*_V_(*T*) vs. *T* data. The difference in *J*_V_(*T*) values when using these two equations is up to 3.8% at the warmer temperatures. In future, the new overall fit (i.e., the ln(*J*_V_(*T*)) fit) provided in Table 2 should be used to represent *J*_V_(*T*) from the LOC-NIPI platform. The homogeneous surface nucleation rate coefficient rate, *J*_s_(*T*) (cm^−2^ s^−1^), was not calculated for this data as the literature suggests that, in droplets with diameters greater than approximately 6 μm, nucleation is more likely to occur in the bulk of the droplet than at the droplet surface [7,24,25], while the 86 μm diameter droplets analysed here were far above that range.

Unlike many other droplet assay techniques, each *J*_V_(*T*) value from the LOC-NIPI is generated individually, i.e., without being influenced by the total droplet population of an experiment as a whole. Given this, the data are quite remarkable since more variability would be expected in the former case than the latter (wherein data are calculated cumulatively), but the reproducibility and precision of the data remain high, with the precision being better than 0.2 °C. The three experimental runs were all performed with slightly different parameters (see Table 1), with run 1 intended to be a high resolution analysis comprising many droplets and narrow temperature increments. Run 2 was intended to be a much coarser analysis, with fewer droplets analysed and broader temperature increments, with run 3 sitting between runs 1 and 2 in terms of resolution. The results demonstrated that the much lower resolution analysis of run 3 provided similar overall results to run 1, but in a much shorter timeframe, while the resolutions of runs 1 and 2 provide more confidence in the data collected. Furthermore, the application of slightly different flow rates yielded small variations in droplet sizes between the experimental runs but this also did not affect the *J*_V_(*T*) results.

The LOC-NIPI user can thus operate the platform based on the parameters that they are interested in and the type or quality of the data they wish to collect, with the compromise being the amount of time taken to collect such data. Remarkably, there are very few examples of multiple water droplet freezing runs, and fewer still in homogeneous nucleation studies, being performed in the microfluidics literature [26,33], hence the precision of many microfluidic examples cannot be commented on while it is clearly demonstrated for the LOC-NIPI here.

Due to its continuous flow nature, the LOC-NIPI could be easily adapted to exploring *J*_V_(*T*) at colder and warmer temperatures than shown here in order to expand the experimental range. This would be achieved by vastly increasing the number of droplets analysed per temperature increment, which would allow, for example, the one in thousands or millions of droplets that did not freeze at lower temperatures to be observed. In this case, the possibility of many ice nuclei growing in the micrometre-sized droplets at lower temperatures would need to be considered, as this could affect the detection and interpretation of droplet freezing and potentially lead to an underestimation of *J*_V_(*T*) [52]. Even more care would need to be taken in the temperature measurement as temperatures were lowered, since the temperature accuracy is considered to be the most important uncertainty in *J*_V_(*T*) [26]. Exploring warmer temperatures could be achieved by observing the few droplets that froze out of thousands or millions of liquid droplets, although great care would need to be taken to ensure the purity of the water used so that such freezing events were not due to the presence of impurities [12].

### 3.2. Comparison of the LOC-NIPI to Physically Constrained Classical Nucleation Theory

The LOC-NIPI *J*_V_(*T*) data are initially compared to the parameterisation of Koop and Murray [41], hereafter referred to as KM16, shown as an orange line alongside the LOC-NIPI data in Figure 2b. The KM16 parameterisation is based on a physically constrained version of classical nucleation theory (CNT), in which the diffusion activation energy (based on the translational self-diffusion coefficient of water, *D*) and the stacking-disordered ice–water interfacial energy (*σ*_sd,l_) components were constrained using parameterisations based on experimental data. The LOC-NIPI results compare well with KM16, particularly at warmer temperatures. However, KM16 predicts a significant curvature in the response of *J*_V_(*T*) to temperature, while the LOC-NIPI data yielded a linear response, hence the parameterisation and the LOC-NIPI data diverge at lower temperatures. While it is true that the error bars in temperature overlap with KM16, the uncertainty in the LOC-NIPI temperature is systematic, but the reproducibility indicates the precision is better than 0.2 °C. Hence, it appears that the temperature dependence of *J*_V_(*T*) is inconsistent with KM16. 

This discrepancy may lie in some of the fittings and correlations employed by Koop and Murray in deriving their parameterisation. The only adjustable parameter in KM16 is the absolute value of *σ*_sd,l_ (18.5 mJ m^−2^) at a reference temperature of 236 K (−37.15 °C), which is scaled with temperature using the Turnbull correlation. However, extrapolation of *σ*_sd,l_ to the melting temperature of ice (273.15 K; 0 °C) yielded a *σ*_sd,l_ that was on the lower end of what may be expected from other literature, hence the reference value of *σ*_sd,l_ used may be too low. Further, the Turnbull correlation produces a stronger temperature dependence of *σ*_sd,l_ than other methods, which in turn strongly influences the temperature response of *J*_V_(*T*) and could contribute to the sharp curve of the *J*_V_(*T*) parameterisation. Finally, the type of fit applied to the *D* data can also greatly affect the steepness and curvature of the *J*_V_(*T*) parameterisation, as demonstrated in Koop and Murray [41] who showed multiple viable fits and their effect on *J*_V_(*T*). *D* is unconstrained by data below ~238 K (−35 °C), hence the poor agreement between theory and this new data may indicate that the extrapolation of this quantity should be revisited.

A similar argument can also be made for the treatment of the vapour pressure of supercooled liquid water, *P*_l_, used in KM16, which is based on the parameterisation of Murphy and Koop [53] (see Equation (A2) in Appendix B). Precise measurements of *P*_l_ are available down to approximately 260 K (−13.15 °C), below which supercooled water is treated as being in thermodynamic continuity with amorphous solid water (ASW) at ~155 K (−118.15 °C), the temperature below which ASW can typically be measured, allowing an estimation of *P*_l_ in the range of 123 K to 332 K (−150.15 to 58.15 °C). However, Nachbar et al. [54,55] recently reported higher than expected vapour pressures of ASW between 133 and 147 K (−140.15 to −126.15 °C), questioning the assumption that supercooled liquid water and ASW belong to the same phase. Therefore, it may not be appropriate to produce a vapour pressure curve connecting the two, hence the estimation of *P*_l_ below (for supercooled water) and above (for ASW) the temperature ranges of the available experimental data are subject to a high degree of uncertainty. The effect may not be large around the homogeneous nucleation range shown in Figure 2b, but would certainly have an effect and could further explain some of the discrepancies.

In summary, KM16 is sensitive to the fit of *D*, the reference value of *σ*_sd,l_, and the extrapolation of *σ*_sd,l_, each of which could be tweaked in a realistic manner to provide various viable representations of *J*_V_(*T*). It is further potentially subject to a high level of uncertainty in the estimation of *P*_l_. While our measurements challenge the exact values used in KM16, more information is needed to determine which values should be used instead. This could be addressed by exploring *J*_V_(*T*) at warmer and lower temperatures using micrometre-sized droplets in order to determine, for example, which fit of *D* provides the best fit of *J*_V_(*T*) to experimental data across a wider temperature range, thus helping to further constrain the parameterisation. As described in the previous section, the LOC-NIPI could provide a route to achieving this thanks to the potential to look for the few droplets in thousands or millions that do or do not freeze at these extremes.

### 3.3. Comparison of Microfluidic Volume Nucleation Rate Coefficient, J_V_(T), Values in the Literature

**Figure 3:**
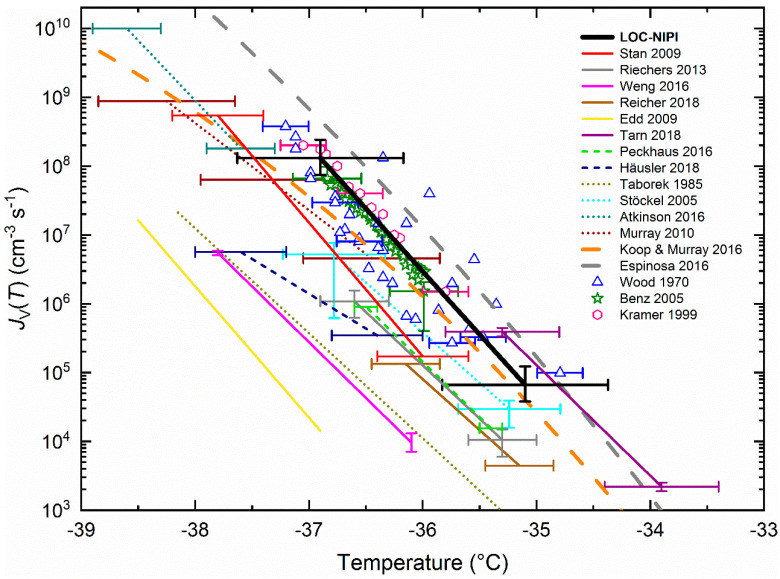
The volume nucleation rate coefficient, *J*_V_(*T*), values obtained using the LOC-NIPI, alongside data from microfluidic platforms in the literature (solid lines) [26,27,28,29,31,32,33,37]. Parameterisations calculated for Peckhaus et al. [31] and Häusler et al. [33] in this article are shown as dashed lines. A selection of non-microfluidic parameterisations [7,49,51,62] (shown as dotted lines) and datasets (shown as discrete points) [59,60,61] are also provided. The physically constrained CNT parameterisation of Koop and Murray [41] and a parameterisation based on the TIP4P/Ice model by Espinosa et al. [14,63] are shown as broadly dashed lines. Details of all of the microfluidic parameterisations can be found in Table 3.

The LOC-NIPI parameterisation for *J*_V_(*T*) has been compared to a selection of datasets and a variety of parameterisations available in the literature in Figure 3. In particular, we briefly describe the various microfluidic techniques that have been employed for the freezing of pure water droplets and have compiled the details of the *J*_V_(*T*) parameterisations (including some that we have calculated here from the literature data) in Table 3. This allows us to not only compare the LOC-NIPI to other contemporary microfluidic data, but also as a means of comparing microfluidically generated data to commonly used homogeneous freezing data and parameterisations as a whole. This summary of the literature also serves as a prelude to our later discussion of the stacking-disordered ice–water interfacial energy, wherein the compiled parameterisations were used to calculate values of *σ*_sd,l_, a parameter that has rarely been explored in microfluidics.

The microfluidic instruments listed in Table 3 have utilised several different techniques for studying the freezing of pure water, and we have grouped these techniques into three categories for discussion depending on the level of complexity: (i) on-chip droplet generation with off-chip freezing, (ii) on-chip microarray freezing, and (iii) freezing in continuous flow. It is worth noting, however, that many examples involve the freezing of purified water as a baseline for INP studies, with only a handful focussing on the study of homogeneous freezing [26,29,37,38], hence we provide a little more discussion for these latter cases given their relevance to this work.

#### 3.3.1. On-Chip Droplet Generation with Off-Chip Freezing

The simplest technique involves the microfluidic generation of droplets for collection, with their subsequent off-chip freezing as an emulsion of monodisperse water-in-oil droplets. The basic microfluidic setup required, the ability to use a conventional cold stage for the cooling of droplets, and the simplicity of the method makes it highly accessible for research groups. However, the technique is more manually intensive than more complex microfluidic methods, and the number of droplets analysed per experiment is limited (i.e., hundreds of droplets), limiting the statistics compared to techniques that can analyse thousands of droplets per experiment. Further, surfactant is required to prevent the coalescence of the droplets following their generation and collection since the droplets are in contact with each other. However, after freezing and then thawing, the droplets can lose their stability and coalesce, hence freeze–thaw experiments cannot typically be performed using this format.

This emulsion technique was first demonstrated by Riechers et al. [26] in their study of homogeneous nucleation, in which they used differential scanning calorimetry (DSC) to determine *J*_V_(*T*) with high temperature accuracy over a range of micrometre-scale droplet diameters. This allowed them to demonstrate how temperature accuracy is the most important uncertainty in the determination of *J*_V_(*T*), while studying multiple droplet size ranges yielded good precision. However, the range of *J*_V_(*T*) values explored was small (see Figure 3), and it was unclear how many droplets were analysed in each DSC run. Weng et al. [27] froze water-in-oil emulsions as part of a cryobiology-oriented study involving heavy water (deuterium oxide, D_2_O), Snomax (a highly active INP type), and cryoprotectants. Parameterisations of *J*_V_(*T*) were generated for H_2_O and D_2_O as part of the overall study, albeit from only single runs of each. Although modelling of the temperature lag between the droplets and the cold plate was performed, no temperature uncertainty or calibration details were provided, which may explain why the *J*_V_(*T*) parameterisation covers a colder temperature range than may be expected given other literature (see Figure 3). Tarn et al. [28] developed a microfluidic version of the picolitre Nucleation by Immersed Particle Instrument (pL-NIPI) by generating monodisperse droplets with a microfluidic device, before freezing them on a Peltier element-based cryomicroscope stage for the study of atmospheric INPs. Previously, the pL-NIPI had involved the nebulisation of polydisperse pL droplets onto a hydrophobic slide for freezing via a liquid nitrogen-controlled cryomicroscope stage [7,49]. A *J*_V_(*T*) parameterisation from a single pure water dataset was provided as part of the study, but was not taken any further in terms of homogeneous nucleation analysis.

#### 3.3.2. On-Chip Microarray Freezing

By increasing the complexity of the microfluidic device, it is possible to provide a more automated droplet generation and freezing process. Rather than generating and collecting droplets for off-chip analysis, droplet traps can be fabricated within the chip itself, allowing the trapping of droplets in an array, with the entire chip being subsequently frozen. This negates the need for the manual transfer of droplets to the cold stage, since the droplets can be generated, trapped, and frozen all within the one device. The use of a regular array of droplets also makes the analysis easier to automate, while trapping the droplets in separate locations allows freeze–thaw experiments to be performed without the risk of droplets coalescing following their initial thawing. However, the design and fabrication of the device requires more microfluidic expertise than the simple emulsion method described in Section 3.3.1, and the method is limited to hundreds of droplets per experiment hence the statistics of the technique can suffer in comparison to instruments that can analyse thousands of droplets.

Edd et al. [29] developed a microfluidic array of droplets using a “Dropspots” [56] device, and froze the droplets on-chip using a commercial cryomicroscope stage in one of the first microfluidic studies of homogeneous nucleation. In addition to providing an early microfluidic *J*_V_(*T*) parameterisation, the authors demonstrated the first instance of using microfluidics to calculate an ice–water interfacial energy, based on the nucleating phase being hexagonal ice (i.e., *σ*_h,l_, as opposed to *σ*_sd,l_ discussed throughout this article), of 33.4 mJ m^−2^. However, no uncertainties or details of temperature calibration were provided, and the *J*_V_(*T*) parameterisation appears in a lower temperature range than would be expected given other literature (see Figure 3). Reicher et al. [32] later developed a new platform based on a Dropspots array for the study of INPs, known as the “WeIzmann Supercooled Droplets Observation on a Microarray” (WISDOM). This included an extensive temperature calibration, and the freezing of a population of water droplets as part of the study brought this Dropspots-based *J*_V_(*T*) parameterisation in line with the available literature (see Figure 3).

Some works in the microfluidics literature are related to the above emulsion and array techniques, but are designed and operated in different manners. Peckhaus et al. [31] used a commercial piezoelectric actuator-based microfluidic droplet generator to print an impressive 1500 droplets onto a cold stage for INP analysis. No *J*_V_(*T*) was provided since the article was focussed on the study of ice nucleation on mineral particles, but we have digitised their *f*_ice_(*T*) data for purified water in order to calculate *J*_V_(*T*), albeit only from data in the coldest, steepest part of the *f*_ice_(*T*) curve, and this new parameterisation is provided in Table 3. We have also performed this digitisation and parameterisation process for the work of Häusler et al. [33], who demonstrated “Freezing on a chip” for the study of INPs via the generation of droplets in an array of microcavities on a cold stage. However, the chip was limited to tens of droplets, and the method of droplet generation required several manual steps. These new *J*_V_(*T*) parameterisations for the Peckhaus et al. [31] and Häusler et al. [33] data are plotted in Figure 3 as dashed lines.

Other examples of the freezing of purified water in a microfluidic droplet array are also available from which *J*_V_(*T*) data are not provided, but should nonetheless be made aware of. Sgro et al. [30] demonstrated a droplet docking array that allowed the environment around the trapped frozen droplets to be exchanged for different immiscible phases, but this device was not employed for the study of homogeneous freezing. Recently, Brubaker et al. [34] developed a “store and create” [57] microfluidic droplet array to freeze 6 nL droplets for the analysis of INPs, and was used by the authors as part of an overview of best practices for the freezing of pure water droplets [12]. However, they refrained from identifying their purified water results as “homogeneous” freezing [34], hence we have not included their data here.

#### 3.3.3. Droplet Freezing in Continuous Flow

The most complex form of microfluidic ice nucleation thus far involves the generation and freezing of droplets in continuous flow as they pass through a cold region within the microchannel, such as in the LOC-NIPI. The exact methods employed can vary in difficulty, and the measurement of the droplet temperature becomes more complicated than in the previously discussed techniques since the droplets are in flow. Further, the size increase in a water droplet freezing to an ice crystal (a 9% increase in volume), in addition to the possible formation of spicules (protuberances of frozen water that can extrude from a droplet [58]), mean that the channel geometry must be sufficiently larger than the droplets such that they do not become stuck after freezing, whilst keeping the channel dimensions relatively small to improve heat transfer. However, if these challenges can be overcome, continuous flow platforms can provide a route to full automation of the ice nucleation analysis procedure, with the potential for analysing thousands or millions of droplets per experiment, as required by the user.

Sgro et al. [36] demonstrated the first example of freezing droplets in flow, but did not perform a droplet freezing analysis, hence data are unavailable for a parameterisation of *J*_V_(*T*). Stan et al. [37,38] developed an elegant device in which flowing droplets passed through a defined temperature gradient, with the position at which the droplets froze equating to the freezing temperature. This allowed the high-throughput and automated analysis of over 37,000 individual droplet freezing events, but required a sophisticated microfabricated setup for performing in-channel temperature measurements, which further required temperature modelling of the flowing droplets. While Stan et al. do not provide a parameterisation of *J*_V_(*T*), we have used Equation (3) to calculate an equation based on their *J*_V_(*T*) data, and this is provided in Table 3 and Figure 3. This platform was also later used by Stan et al. [38] to study the effect of electric fields on homogeneous freezing, finding that fields up to at least 1.6 × 10^5^ V m^−1^ did not influence ice nucleation.

#### 3.3.4. Non-Microfluidic Examples for Comparison

Alongside the microfluidic parameterisations discussed above, a handful of relevant non-microfluidic *J*_V_(*T*) parameterisations and datasets are also provided in Figure 3. Murray et al. [49] used the original pL-NIPI technique to study the homogeneous freezing of polydisperse droplet populations (10 to 40 μm diameter), and the resultant *J*_V_(*T*) parameterisation is shown in Figure 3 as a dotted line. The wide droplet size bins employed by Murray et al. led to a shallow bias in the temperature dependence of *J*_V_(*T*). The authors also used their data to determine the stacking-disordered ice–supercooled water interfacial energy, *σ*_sd,l_, a characteristic we discuss in detail later in this article. They further calculated and provided *σ*_sd,l_ values from the homogeneous freezing data of other publications, including Wood and Walton [59], Benz et al. [60], Kramer et al. [61], as well as the microfluidic data of Stan et al. [37]. Since since we use these *σ*_sd,l_ values later in this article we have also included the relevant *J*_V_(*T*) datasets in Figure 3 (shown as symbols). Atkinson et al. [7] also used the original pL-NIPI technique to generate 4 to 24 μm diameter droplets, binned into much smaller size ranges, via nebulisation for freezing, and used these droplets to study the relative contribution of volume vs. surface nucleation at different droplet volumes as mentioned earlier. The *J*_V_(*T*) parameterisation from this work is also provided in Figure 3 as a dotted line.

Two parameterisations that have commonly been used for comparisons in microfluidic homogeneous freezing literature, and hence have been included here, are from Taborek et al. [62], who used an adiabatic calorimeter to study the freezing of droplet emulsions of a range of micrometre-scale diameters, and Stöckel et al. [51], who froze individual levitated droplets (~80-90 μm diameter) in an electrodynamic balance. Finally, we also provide the physically constrained CNT parameterisation of Koop and Murray [41] (KM16), as described previously in Section 3.2, alongside a parameterisation by Espinosa et al. [63] (fit obtained from Amaya et al. [14]) based on the TIP4P/Ice model [64], both of which are shown as broadly dashed lines in Figure 3. We further note that the data and parameterisations presented in Figure 3 are not exhaustive, and more detailed reviews of datasets can be found in, for example, Ickes et al. [6] and Atkinson et al. [7].

#### 3.3.5. Comparisons to the LOC-NIPI

The available literature microfluidic *J*_V_(*T*) data fall between approximately 3 × 10^3^ and 4 × 10^8^ cm^−3^ s^−1^, within a temperature range of −33.9 to −38.5 °C (a range of 4.6 °C) at its extremes, which is likely due to the lack of temperature calibration in some of the early examples, while the bulk of the data lie between approximately −35.1 and −37.8 °C (a range of 2.7 °C). The LOC-NIPI data, comprising over 16,000 droplets and high precision across three experimental runs, compares well with, if at slightly warmer temperatures than, much of the other microfluidic data, and in particular compares very well with data from some of the non-microfluidic datasets [59,60,61]. A noteworthy feature of the LOC-NIPI data is that it covers a wide *J*_V_(*T*) range, being much larger than most of the microfluidic emulsion and on-chip microarray techniques due to the greater number of droplets analysed, with only the other continuous flow technique of Stan et al. [37] being comparable. This is promising for the LOC-NIPI since the numbers of droplets analysed at each temperature step could easily be increased to push the data into warmer and cooler temperatures.

Like the LOC-NIPI data described in Section 3.2, neither the microfluidic nor the non-microfluidic examples exhibit the curvature present in KM16 [41], but the data in general fall close to this *J*_V_(*T*) line. The steepness of the LOC-NIPI line is also similar to some of the more recent microfluidic and non-microfluidic examples that have often had additional care taken in temperature calibration compared to some older examples. The TIP4P/Ice parameterisation of Espinosa et al. [14,63] predicts *J*_V_(*T*) at warmer temperatures than KM16 and most of the data shown but remains reasonably close to the LOC-NIPI and several other datasets. It also exhibits less curvature than KM16, being more in line with the more recent experimental datasets.

Although there is still some spread in the data from the microfluidic and non-microfluidic sources, it is also limited to a relatively small range of *J*_V_(*T*) values. It is extremely promising that the majority of the microfluidic data, particularly those with more detailed descriptions of temperature calibration, appear in the same *J*_V_(*T*) region between the different techniques, but also in the same region as the previous non-microfluidic data. With further work, including the generation of datasets with large numbers of droplets and the careful measurement of temperature [26], it may be possible to reduce this spread and so help to inform new *J*_V_(*T*) parameterisations. In particular, it would be beneficial to greatly expand on the *J*_V_(*T*) range by employing microfluidic devices to observe freezing events at both much warmer and much colder temperatures as mentioned previously, since data in these regimes are relatively sparse and very varied [14,15,16,17,18,19,20,21,22]. In addition, some of the data at lower temperatures (≲−40 °C, not shown here) are based on nanometre-sized droplets (but not always [15,65]) that have large internal Laplace pressures that may influence the rate of nucleation compared to droplets at ~1 bar. This probing into other temperature regimes could be achieved with many of the available microfluidic platforms, although droplet emulsion and microarray techniques would struggle, at least without the time-consuming manual resetting of experiments, to observe single events out of thousands or millions of droplets. However, continuous flow droplet freezing platforms such as the LOC-NIPI [39] and the platform of Stan et al. [37,38] are much better suited to this type of study since they can be allowed to run for as long as the user requires in order to gather the necessary data.

Thus, as discussed earlier, the LOC-NIPI could become a very powerful tool in the study of homogeneous freezing at much lower and even warmer temperatures than is typically explored. In order to achieve this, the temperature uncertainty would need to be reduced, which could be achieved by incorporating features into the chip to ensure reproducible alignment of the channel on the cold plate. Further, an additional temperature reference channel could be added in order to provide temperature measurements on either side of the droplet freezing channel, and these reference channels could be moved closer to the droplet channel in order to ensure high temperature accuracy. Slight alterations to the chip design would be required to limit the temperature gradient experienced by the droplets as they pass over the cold plate, e.g., the incorporation of a serpentine channel to increase the time spent at a defined temperature. The use of syringe pumps would also need to be replaced with pumps more amenable to achieving continuous flow over long periods of time, e.g., peristaltic pumps, while automated analysis would be required in order to analyse the large number of droplets necessary for such experiments.

### 3.4. Interfacial Energy of the Stacking-Disordered Ice–Supercooled Water Interface, σ_sd,l_

Given the tight spread and linearity of the LOC-NIPI *J*_V_(*T*) dataset obtained from >16,000 droplets, we were able to use this data to estimate the interfacial energy of the stacking-disordered ice cluster–supercooled water interface (i.e., the amount of energy per unit area required to create a surface of ice from water), *σ*_sd,l_, according to classical nucleation theory (CNT), using the method described in detail by Murray et al. [49]. The interfacial energy is an important component in the nucleation rate of homogeneous freezing, as discussed in Section 3.2. This calculation has previously not been performed as part of any other microfluidic homogeneous nucleation study, with the only other relevant examples being a value determined by Murray et al. [49] from the microfluidic dataset of Stan et al. [37], and a value of the hexagonal ice–supercooled water interfacial energy (*σ*_h,l_) by Edd et al. [29].

The ice cluster is thought to have a stacking-disordered structure (ice I_sd_), where cubic (ice I_c_) and hexagonal (ice I_h_) sequences are interlaced [66,67], and has a higher free energy than the most stable hexagonal phase [41]. CNT describes the Gibbs energy required to generate an ice cluster of critical size, Δ*G*_crit_, from which the cluster can grow into a crystal, according to Equation (4) [41,49]:

(4) $$\Delta G_{\mathrm{crit}}=\frac{16\;\pi \;v^{2}\;\sigma_{\mathrm{sd},l}^{3}}{3{\left(k\;T\;\left(\ln S\right)\right)}^{2}}$$

where *v* is the volume of a water molecule in ice (3.24 × 10^−29^ m^3^), *σ*_sd,l_ is the interfacial energy between ice I_sd_ and supercooled liquid water, and *k* is the Boltzmann constant. *S* is the saturation ratio with respect to the nucleating phase of ice, assumed to be ice I_sd_ [41]:

(5) $$S=\frac{P_{l}}{P_{\mathrm{sd}}}=\frac{P_{l}}{P_{h}\;\exp\left(\frac{\Delta G_{h\to \mathrm{sd}}}{R\;T}\right)}$$

where *P*_l_ is the vapour pressure of supercooled water, *P*_sd_ is the vapour pressure of ice I_sd_, *P*_h_ is the vapour pressure of ice I_h_, *R* is the gas constant, and Δ*G*_h__→__sd_ is the free energy of the transformation of ice I_h_ to ice I_sd_. *P*_sd_ was calculated as a function of temperature assuming Δ*G*_h__→__sd_ = 155 ± 30 kJ mol^−1^ at 185 K [68], and using *P*_h_ values calculated as per Murphy and Koop [53] (see Equation (A1) in Appendix B). *P*_l_ was calculated as a function of temperature as per Murphy and Koop [53] (see Equation (A2) in Appendix B), and was used with *P*_sd_ to determine *S* according to Equation (5). However, it must be borne in mind that the estimation of *P*_l_ is subject to the same uncertainties as described in Section 3.2, given the recent findings of Nachbar et al. [54,55]. 

The volume nucleation rate coefficient, *J*_V_(*T*), is related to Δ*G*_crit_ in an Arrhenius-style equation, where *A* is a pre-exponential factor:

(6) $$J_{V}\left(T\right)=A\;\exp\left(-\frac{\Delta G_{\mathrm{crit}}}{k\;T}\right)$$

Combining Equations (4) and (6) gives [49]:

(7) $$\ln\;J_{V}\left(T\right)=\ln\;A\;-\frac{16\;\pi \;v^{2}\;\sigma_{\mathrm{sd},l}^{3}}{3\;k^{3}\;T^{3}\;{\left(\ln S\right)}^{2}}\;$$

Thus, by plotting our *J*_V_(*T*) data in terms of ln *J*_V_(*T*) vs. *T*^−3^ (ln *S*)^−2^, the resultant linear fit (*y* = *mx* + *c*) has an intercept, *c*, of ln *A* and a slope, *m*, of:

(8) $$m=-\frac{16\;\pi \;v^{2}\;\sigma_{\mathrm{sd},l}^{3}}{3\;k^{3}}$$

**Figure 4:**
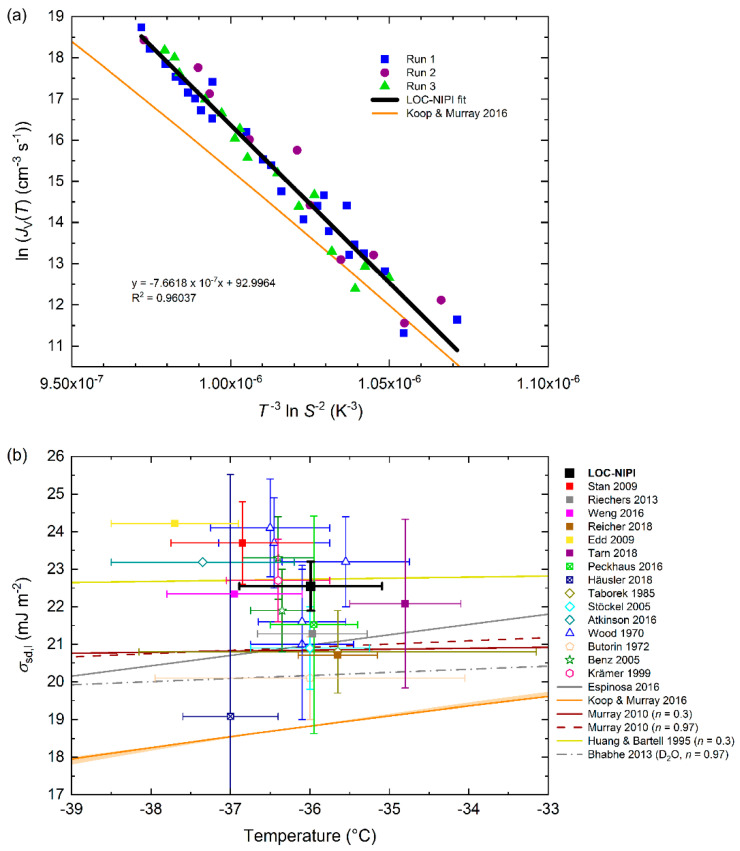
Determination of the interfacial energy of stacking-disordered ice–supercooled water, *σ*_sd,l_. (**a**) The LOC-NIPI nucleation rate data as a function of *T*^−3^ (ln *S*)^−2^. The slope of the linear fit corresponds to *σ*_sd,l_ = 22.5 ± 0.7 mJ m^−2^, with an intercept corresponding to *A* = 2.443 × 10^40^ cm^−3^ s^−1^. The parameterisation of Koop and Murray is shown for comparison [41]; (**b**) comparison of the LOC-NIPI derived interfacial energy to those determined from other microfluidic instruments (shown as solid squares) [26,27,28,29,32,37], including those calculated from digitised data (shown as squares with crosses inside) [31,33]. In addition, parameterisations available in the literature (shown as lines) [14,23,41,49,63,70,71,72], a value calculated here from Atkinson et al. [7], and literature values [7,51,59,60,61,62,69] (shown as various symbols) obtained from Murray et al. [49] are presented. Symbols represent the midpoint of the temperature range explored in each case, while the error bars represent the full temperature range that the *σ*_sd,l_ data covered. Parameterisations of Murray et al. [49], Huang and Bartell [23], and Bhabhe et al. [70] based on a temperature dependency of *σ*_sd,l_ were determined using the equation *σ*_sd,l_ = *σ*_sd,l_(*T*_0_) × (*T*/*T*_0_)*^n^* [23], where *T* is temperature and *σ*_sd,l_(*T*_0_) is a reference interfacial energy at reference temperature *T*_0_. Values of *σ*_sd,l_(*T*_0_), *T*_0_, and *n* (provided in the legend) were obtained from the relevant publications.

This plot is shown in Figure 4a for the LOC-NIPI data, with the intercept yielding: ln *A* = 92.996 (hence *A* = 2.443 × 10^40^ cm^−3^ s^−1^), and the slope yielding: *m* = −(7.662 ± 0.222) × 10^7^ K^3^. Figure 4a also shows the ln *J*_V_(*T*) vs. *T*^−3^ (ln *S*)^−2^ values from the parameterisation of Koop and Murray [41], to which the LOC-NIPI data follow a similar trend but deviate at lower values of *T*^−3^ (ln *S*)^−2^, again showing a curvature not exhibited by the experimental data.

Since *v* and *k* are known, the interfacial energy of stacking-disordered ice–supercooled water, *σ*_sd,l_, can thus be calculated from *m* via Equation (9):

(9) $$\sigma_{\mathrm{sd},l}=\sqrt[{3}]{-\left(\frac{3\;k^{3}\;m}{16\;\pi \;v^{2}}\right)}$$

This gives an interfacial energy of *σ*_sd,l_ = 22.5 ± 0.7 mJ m^−2^, with the uncertainty determined from the standard error of the slope, *m*, as per Murray et al. [49]. The value of *σ*_sd,l_ from the LOC-NIPI is plotted in Figure 4b against literature values [37,51,59,60,61,62,69] (previously described in Section 3.3 in terms of *J*_V_(*T*)) taken from data compiled by Murray et al. [49], in addition to a value that we have calculated from the pL-NIPI *J*_V_(*T*) parameterisation of Atkinson et al. [7] (23 mJ m^−2^). Further to this, we have also used Equations (7)–(9) to determine values of *σ*_sd,l_ from all of the microfluidic techniques used to study homogeneous freezing. Ideally, this should be done using original datasets, but in most cases here we have had to calculate the values from the *J*_V_(*T*) parameterisations in Table 3 [26,27,29,32,37], and these are shown as solid squares in Figure 4b. We note that the values obtained from *J*_V_(*T*) parameterisations do not have *σ*_sd,l_ error bars. Values obtained from original or digitised datasets [28,31,33] are shown as squares with a cross inside in Figure 4b. The calculated microfluidic values of *σ*_sd,l_ and ln *A* are provided in Table 3. The value for Stan et al. [37] was taken from Murray et al. [49], who determined it using the original *J*_V_(*T*) data. The temperature error bars in Figure 4b represent the range of temperatures covered in each piece of work, rather than the temperature uncertainties.

The bulk of the *σ*_sd,l_ values, both microfluidic and non-microfluidic, shown in Figure 4b cover a range of approximately 21–24 mJ m^−2^, as shown in Figure 4b. Ickes et al. [6] also summarised a great deal of available literature data, and showed that the majority appeared in the 20–25 mJ m^−2^ range at the temperatures of interest here. Thus, the LOC-NIPI value of 22.5 ± 0.7 mJ m^−2^ falls in the middle of this range, demonstrating excellent comparison to the literature, and most notably with a much narrower uncertainty compared to other original datasets. This feature of the LOC-NIPI data is even more apparent when compared to the few datasets in which multiple experiments were performed and yielded very wide distributions [59,60].

Several interfacial energy parameterisations [14,23,41,49,63,70,71,72] are also shown in Figure 4b for comparison with the LOC-NIPI and other microfluidic techniques. The Koop and Murray [41] parameterisation (KM16) for *σ*_sd,l_ was discussed earlier (see Section 3.2). The points raised in the previous discussion of KM16 are also demonstrated here, in particular a very steep temperature dependence and a lower value of *σ*_sd,l_ than may be expected given the other data shown, and the potential reasons for this are described in Section 3.2.

The LOC-NIPI data sit very close to a parameterisation based on the data of Huang and Bartell [23], who froze clusters of water at ~200 K (−73.15 °C) and showed that it was not the ice I_h_ phase that nucleated (stating that it was the ice I_c_ phase that nucleated, but the patterns were actually consistent with the ice I_sd_ phase). This line is actually close to a great deal of the available experimental data, much of which was not available at the time of the original publication.

Parameterisations of the data obtained using the original pL-NIPI by Murray et al. [49], whose methods of interfacial energy analysis are used in this article, fall between the Koop and Murray line and the Huang and Bartell line [23]. These parameterisations are closer to the bulk of the experimental data shown, albeit slightly lower in terms of *σ*_sd,l_. The *σ*_sd,l_ value determined here from the data of Atkinson et al. [7], also obtained using the original pL-NIPI, was higher than Murray et al. [49] due to the analysis of far more droplets and much tighter size binning that resulted in a steeper slope of the *J*_V_(*T*) curve compared to Murray et al. Therefore, the more recent value for Atkinson et al. [7], to which the LOC-NIPI data are closer, is assumed to be more accurate than that of Murray et al. [49]. A parameterisation of Bhabhe et al. [70] for heavy water (D_2_O) is shown in Figure 4b for reference, although this cannot be directly compared but is included here as in other comparative studies [41]. The TIP4P/Ice model parameterisation of Espinosa et al. [63,71,72] (fit obtained from Amaya et al. [14]) falls within a similar region to that of Murray et al. [49], albeit with a much steeper slope, and so is close to, but at the lower end of, the bulk of the experimental data.

The *σ*_sd,l_ value for the microfluidic data of Edd et al. [29] was determined to be 24.2 mJ m^−2^ using the method described in Equations (7)–(9) (as shown in Table 3), based on the *J*_V_(*T*) parameterisation provided by the authors. However, the authors provide an ice–water interfacial energy value of 33.4 mJ m^−2^ in their original article, which is a large difference to the one that we show here. Further, Edd et al. compared their work to Stöckel et al. [51] by calculating a value of 29.2 mJ m^−2^ for the latter, whereas Murray et al. [49] determined a value of 20.9 ± 1.1 mJ m^−2^ for Stöckel et al. using Equations (7)–(9). Edd et al. do not specifically state which phase of ice is considered to nucleate in their interpretation of the ice–water interfacial energy calculations, but we assume from their work that it was hexagonal ice (ice I_h_), i.e., giving *σ*_h,l_, whose values are expected to be larger than those of *σ*_sd,l_. Therefore, given that Ickes et al. [6] also showed a majority of data appearing in the 20–25 mJ m^−2^ range at the temperatures of interest here, we are confident that our value shown in Table 3 and Figure 4b for Edd et al. [29] provides a suitable representation of *σ*_sd,l_, while the authors’ original value of 33.4 mJ m^−2^ represents *σ*_h,l_.

The LOC-NIPI allowed the calculation of a *σ*_sd,l_ value with a very small uncertainty compared to values in the literature (based on original data rather than parameterisations), achieved via the analysis of >16,000 monodisperse droplets across three separate experiments with a very tight distribution of the data. The ability to obtain reproducible data with low uncertainties will be highly beneficial for determining *σ*_sd,l_ values above and below the temperature ranges demonstrated in Figure 4b, as described earlier, in order to extend and constrain future parameterisations.

## 4. Conclusions

We have studied the homogeneous nucleation of water using data from >16,000 monodisperse droplets using the continuous flow LOC-NIPI platform. The freezing characteristics of the droplets were highly reproducible across three runs, allowing the calculation of the homogeneous nucleation rate coefficient, *J*_V_(*T*), in the range of −35.1 to −36.9 °C, and covering a wider range of *J*_V_(*T*) values than in most other examples. Notably, we have taken these results further than in other microfluidic studies by estimating the stacking-disordered ice–water interfacial energy, *σ*_sd,l_ (22.5 ± 0.7 mJ m^−2^), with smaller uncertainties than comparable literature data.

Further to the analysis of the LOC-NIPI data for *J*_V_(*T*) and *σ*_sd,l_, we have also compiled and, where necessary, calculated these from all of the available microfluidic techniques in the literature that provide purified water freezing data. In addition to providing a single source from which to obtain a range of literature microfluidic parameterisations and values, these allowed for comparisons to the LOC-NIPI results, and to compare microfluidic data in general to commonly used parameterisations and datasets from non-microfluidic methods. The LOC-NIPI data compared favourably with the bulk of the microfluidic and non-microfluidic data in terms of both *J*_V_(*T*) and, in particular, *σ*_sd,l_, with most of the microfluidic *σ*_sd,l_ data providing values in the 21–24 mJ m^−2^ range. This is a higher range than expected by several parameterisations, but falls within a similar range to a number of non-microfluidic datasets. This suggests that there remains scope for determining parameterisations that truly represent the experimentally observed properties of purified water. This remains particularly true at temperatures both higher and lower than the mid −30 °C range, with only a handful of highly variable datasets available at much lower temperatures that were largely obtained using nanometre-scale droplets having large internal Laplace pressures and a high probability of surface rather than volume nucleation.

Comparison of the LOC-NIPI *J*_V_(*T*) data with a recent theory-based parameterisation [41] reveals that the curvature in the parameterisation is inconsistent with our high-precision data. This indicates that there may be an issue with the representation of the self-diffusion coefficient of supercooled water, the representation of the interfacial energy, or the vapour pressure of supercooled water (or a combination of these factors). Accurate and precise measurements of the rate of homogeneous nucleation may provide a means of probing fundamental physical properties of deeply supercooled water that are not readily accessible through other techniques in order to help further constrain theoretical representations.

Given the quality of the LOC-NIPI data in the −35.1 to −36.9 °C range, with low *J*_V_(*T*) and *σ*_sd,l_ uncertainties and high reproducibility, this platform could provide a new route to probing homogeneous freezing at higher and lower temperatures using micrometre-scale droplets at atmospheric pressure. Thanks to the continuous flow nature of the platform, it should be possible to analyse thousands or millions of droplets at set temperatures and observe the small number of droplets that do or do not freeze. Should such studies be successful, it would greatly improve our understanding of *J*_V_(*T*) and *σ*_sd,l_, and allow the development of new parameterisations to cover a wider temperature range with smaller uncertainties than is currently available.

## Figures and Tables

**Table 1:** Experimental parameters and droplet characteristics for three experimental runs for studying the homogeneous freezing of purified water droplets.

Run.	Water Flow Rate (μL min^−1^)	Oil Flow Rate (μL min^−1^)	Droplet Generation Rate (droplets s^−1^)	Total No. of Droplets	Droplet Diameter (μm)	Droplet Volume (pL) (× 10^−9^ cm^3^)	Droplet Velocity (mm s^−1^)	Temp. Increment (°C)	No. of Droplets per *T* Increment	Approx. Total Volume (μL)
1	0.05	22	2.0 ± 0.5	10,881	84 ± 7 (CV = 8%)	311 ± 76	10.9 ± 0.1	0.1	403 ± 116	3.38
2	0.05	24	1.6 ± 0.6	1,833	85 ± 7 (CV = 8%)	317 ± 73	11.9 ± 0.1	0.2	167 ± 62	0.58
3	0.02	24	2.1 ± 0.4	3,692	89 ± 7 (CV = 8%)	371 ± 85	11.9 ± 0.1	0.1	217 ± 48	1.37
**Overall**				**16,406**	**86 ± 8**	**331 ± 89**				**5.33**

**Table 2:** Parameterisations of the volume homogeneous ice nucleation rate coefficient, *J*_V_(*T*), for each of the three experimental runs, and the overall parameterisation for all of the data.

Run	Temperature Range (°C)	*J*_V_(*T*) Fit (cm^−3^ s^−1^)	*J*_V_(*T*) Uncertainty (cm^−3^ s^−1^)	R^2^ of *J*_V_(*T*) Fit
1	−35.1 to −36.9	ln *J*_V_(*T*) = −4.0839·*T* – 132.1568	+87%; −43%	0.9582
2	−35.2 to −36.9	ln *J*_V_(*T*) = −4.3261·*T* – 140.7226	+85%; −42%	0.9325
3	−35.5 to −36.7	ln *J*_V_(*T*) = −4.5820·*T* – 150.2315	+85%; −42%	0.9667
**Overall**	**−35.1 to −36.9**	**ln *J*_V_(*T*) = −4.2171·*T* – 136.9602**	**+87%; −43%**	**0.9528**

**Table 3:** Details of microfluidic techniques used to achieve the homogeneous freezing of water droplets, including parameterisations of the volume nucleation rate coefficient (*J*_V_(*T*)), applicable temperature ranges, uncertainties (where available), and the stacking-disordered ice–supercooled water interfacial energy (*σ*_sd,l_) values. The *σ*_sd,l_ and ln *A* values were calculated here using Equations (7)–(9), as per Murray et al. [49].

Publication/Technique.	Type of Droplet Assay	Droplet Diameter (μm)	Droplet Volume (pL)	Temperature Range (°C)	Temperature Uncertainty (°C)	*J*_V_(*T*) (cm^−3^ s^−1^)	Units of *T* in *J*_V_(*T*) Fit	*J*_V_(*T*) Uncertainty (cm^−3^ s^−1^)	*σ*_sd,l_ (mJ m^–2^)	ln (*A* (cm^–3^ s^–1^))	Refs.
Stan 2009	Continuous flow	80 ± 1	268 ± 10	−36.0 to −37.8	±0.4	ln *J*_V_(*T*) = −4.4746·*T* − 149.0305 ^(a)^	°C	−	23.7 ± 1.1 ^(b)^	102.9 ± 5.0 ^(b)^	[37]
Edd 2009; “Dropspots”	Microfluidic droplet array	37 ± 2	26 ± 5	−36.9 to −38.5	−	log_10_ (*J*_V_(*T*) × 10^−9^) = −1.912·*T* – 75.4	°C	−	24.2 ^(c)^	101.7	[29]
Riechers 2013	Droplet emulsion	53 ± 6 to 96 ± 11	78 ± 30 to 463 ± 178	−35.3 to −36.6	±0.3	ln *J*_V_(*T*) = −3.574·(*T* – 235) + 19.44	K	±43%	21.3	77.4	[26]
Weng 2016	Droplet emulsion	35 ± 2	22 ± 5	−36.1 to −37.8	−	log_10_ *J*_V_(*T*) = (−1.62 ± 0.06)·*T* – (54.5 ± 2.3)	°C	Provided in the *J*_V_(*T*) equation	22.3	84.6	[27]
Peckhaus 2016	Printed droplet array	107 ± 14 (spherical cap)	215 ± 70	−35.4 to −36.5	±0.1	ln *J*_V_(*T*) = −3.6977·*T* – 121.2490 ^(d)^	°C	−	21.5 ± 2.9	79.8 ± 9.1	[31]
Tarn 2018; “Microfluidic pL-NIPI”	Droplet emulsion	94 ± 3	435 ± 43	−33.9 to −35.3	±0.5	log_10_ *J*_V_(*T*) = −1.60674·*T* − 51.12734	°C	±13%	22.1 ± 2.2	88.2 ± 7.6	[28]
Reicher 2018; “WISDOM”	Microfluidic droplet array	100	524	−35.15 to −36.15	±0.3	*J*_V_(*T*) = exp(−3.4·*T* + 817.6)	K	−	20.7	71.8	[32]
Häusler 2018; "Freezing on a Chip”	Microcavity-based droplet array	40 ± 4	34 ± 11	−36.4 to −37.6	±0.4	ln *J*_V_(*T*) = −2.3261·*T* – 71.9161 ^(d)^	°C	−	19.1 ± 6.4	59.1 ± 15.0	[33]
This work; “LOC-NIPI”	Continuous flow	86 ± 8	331 ± 89	−35.1 to −36.9	±0.7	ln *J*_V_(*T*) = −4.2171·*T* – 136.9602	°C	+87%, −43%	22.5 ± 0.7	93.0 ± 2.2	[39] and this work

^(a)^ Parameterisation determined by fitting to the original *J*_V_(*T*) data [37]. ^(b)^ Values obtained from Murray et al. [49]. ^(c)^ Edd et al. [29] reported an interfacial energy of 33.4 mJ m^−2^, most likely the value of *σ*_h,l_, in which hexagonal ice (ice I_h_) is considered to be the nucleating phase. ^(d)^ *J*_V_(*T*) determined by fitting to digitised data of the coldest, steepest part of the purified water *f*_ice_(*T*) curve.

## Data Availability

The datasets for this paper are publicly available in the University of Leeds Data Repository (https://doi.org/10.5518/951, Tarn et al., 2021) [73].

## Appendix B

### Vapour Pressures of Ice and Supercooled Water

To calculate the interfacial energy, *σ*_sd,l_, of the stacking-disordered ice–supercooled water interface, the saturation ratio with respect to the nucleating phase of ice, *S*, was determined using temperature-dependent vapour pressures of hexagonal ice (ice I_h_) and supercooled water. These vapour pressures were obtained using Equations (A1) and (A2), respectively, as per Murphy and Koop [53], where temperature, *T*, has units of K, and pressure, *P*, has units of Pa. The vapour pressure of hexagonal ice, *P*_h_, is shown in Equation (A1) for *T* > 110 K (i.e., *T* > −163.15 °C):

(A1) $$P_{h}=\exp(0.550426-\frac{5723.265}{T}+3.53068\;\ln\left(T\right)-0.00728332\;T$$

The vapour pressure of supercooled water, *P*_l_, is shown in Equation (A2) for 123 < *T* < 332 K (i.e., −150.15 < *T* < 58.85 °C):

(A2) $$\ln\left(P_{l}\right)\;\approx 54.842763-\frac{6763.22}{T}-4.210\;\ln\left(T\right)+0.000367\;T+\tan h\left[0.0415\left(T-218.8\right)\right]\left(53.878-\frac{1331.22}{T}-9.44523\;\ln\left(T\right)+0.014025\;T\right)$$
